# Widespread Discordance of Gene Trees with Species Tree in *Drosophila:* Evidence for Incomplete Lineage Sorting

**DOI:** 10.1371/journal.pgen.0020173

**Published:** 2006-10-27

**Authors:** Daniel A Pollard, Venky N Iyer, Alan M Moses, Michael B Eisen

**Affiliations:** 1 Graduate Group in Biophysics, University of California Berkeley, Berkeley, California, United States of America; 2 Department of Molecular and Cell Biology, University of California Berkeley, Berkeley, California, United States of America; 3 Department of Genome Sciences, Genomics Division, Ernest Orlando Lawrence Berkeley National Lab, Berkeley, California, United States of America; 4 Center for Integrative Genomics, University of California Berkeley, Berkeley, California, United States of America; University of Iowa, United States of America

## Abstract

The phylogenetic relationship of the now fully sequenced species Drosophila erecta and D. yakuba with respect to the D. melanogaster species complex has been a subject of controversy. All three possible groupings of the species have been reported in the past, though recent multi-gene studies suggest that D. erecta and D. yakuba are sister species. Using the whole genomes of each of these species as well as the four other fully sequenced species in the subgenus *Sophophora,* we set out to investigate the placement of D. erecta and D. yakuba in the D. melanogaster species group and to understand the cause of the past incongruence. Though we find that the phylogeny grouping D. erecta and D. yakuba together is the best supported, we also find widespread incongruence in nucleotide and amino acid substitutions, insertions and deletions, and gene trees. The time inferred to span the two key speciation events is short enough that under the coalescent model, the incongruence could be the result of incomplete lineage sorting. Consistent with the lineage-sorting hypothesis, substitutions supporting the same tree were spatially clustered. Support for the different trees was found to be linked to recombination such that adjacent genes support the same tree most often in regions of low recombination and substitutions supporting the same tree are most enriched roughly on the same scale as linkage disequilibrium, also consistent with lineage sorting. The incongruence was found to be statistically significant and robust to model and species choice. No systematic biases were found. We conclude that phylogenetic incongruence in the D. melanogaster species complex is the result, at least in part, of incomplete lineage sorting. Incomplete lineage sorting will likely cause phylogenetic incongruence in many comparative genomics datasets. Methods to infer the correct species tree, the history of every base in the genome, and comparative methods that control for and/or utilize this information will be valuable advancements for the field of comparative genomics.

## Synopsis

To take full advantage of the growing number of genome sequences from different organisms, it is necessary to understand the evolutionary relationships (phylogeny) between organisms. Unfortunately, phylogenies inferred from individual genes often conflict, reflecting either poor inferences or real variation in the history of genes. In this study, the authors examine relationships within the Drosophila melanogaster species subgroup, a group of flies with three fully sequenced species in which phylogeny has been a source of controversy. Although the bulk of the data support a phylogeny with Drosophila melanogaster as an outgroup to sister species Drosophila erecta and *Drosophila yakuba,* large portions of their genes support alternative phylogenies. According to the authors, the most plausible explanation for these observations is that polymorphisms in the ancestral population were maintained during the two rapid speciation events that led to these species. Subsequent to speciation, polymorphisms were randomly fixed in each species, and in some cases non-sister species fixed the same ancestral polymorphisms, while sister species did not. In these cases the genes are correctly inferred to have conflicting phylogenies. The authors note that rapid speciation events will often lead to such conflict, which needs to be accounted for in evolutionary analyses.

## Introduction

With the sequencing of 12 species from the genus *Drosophila,* the field of comparative genomics is now presented with the opportunity and challenge of understanding the function and history of every base in the model organism *Drosophila melanogaster (Dmel).* This process will hopefully result in the discovery of new biological phenomena and the development of new methodologies that will eventually help with the task of annotating other clades in the tree of life, particularly the human genome. Because most analyses of multiple genome sequences involve inferences about evolutionary history, they require an accurate description of the relationship of the species being analyzed.

**Figure 1:**
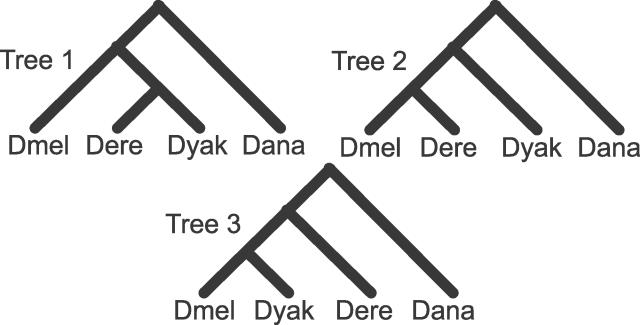
Phylogenies The three possible phylogenies for *Dmel, Dere*, and *Dyak,* with *Dana* as an outgroup.

The species history of the genus *Drosophila* has been the subject of numerous studies, and the consensus from the literature suggests that the relationship of the 12 sequenced species is well resolved, with the exception of the species within the *Dmel* species subgroup and perhaps the placement of the Hawaiian species, *D. grimshawi,* and the *virilis*-*repleta* species, D. virilis and D. mojavenis [1–5]. Within the *Dmel* species group, the placement of *D. erecta (Dere)* and *D. yakuba (Dyak)* relative to the *Dmel* lineage has been the subject of numerous conflicting studies [1–3,6–15]. Considering the placement of *Dmel, Dere,* and *Dyak,* all three of the possible phylogenies (Figure 1) have received support. The topology *(Dmel,(Dere,Dyak)),* which we shall refer to as tree 1, was supported by studies of polytene chromosome banding sequences [6], satellite DNA [7], the *COI* and *COII* mitochondrial genes [3], mitochondrial DNA [16], the *fru* gene [17], the *Cu/Zn SOD* gene [18], the *H3* gene family [19], a concatenation of mitochondrial and nuclear genes [20], a concatenation of the genes *Adh, Adhr, Gld,* and *ry* [8], and a concatenation of the genes *Adh, Amyrel, janA, janB,* and *Sod* [9]. The topology *((Dmel,Dere),Dyak),* which we shall refer to as tree 2, was supported by studies of an internal transcribed spacer region of ribosomal RNA genes [10], nucleotide sequences 5′ of the *Amy* gene [15] and the *Adh* gene [8,21]. The topology *((Dmel,Dyak),Dere),* which we shall refer to as tree 3, was supported by studies of protein electrophoresis [11], mitochondrial DNA [12], single-copy nuclear and mitochondrial DNA hybridization [13], the *Adh* gene [1,14] and the *Amy* gene [15]. The support that each of these studies provides for the three phylogenies, however, is not uniformly strong. The most recent study by Ko et al. using the concatenation of multiple nuclear genes provides the most compelling evidence, with 100% bootstrap support, for the placement of *Dere* and *Dyak* as sister taxa relative to the *Dmel* lineage. That Ko et al. found such strong support for tree 1, despite using the *Adh* gene, which on its own has been found to support the other two trees, suggests that the past incongruence was likely the result of sampling variance [22,23]. Incongruence, however, can also be the result of numerous systematic biases [24–28] that are not overcome by increased sampling [29–31], as well as phylogenetically meaningful phenomena, such as lateral transfer [32] and incomplete linage sorting [25,33–48].

In this study, we set out to examine the possible causes of incongruence in this phylogeny and to investigate the placement of *Dere* and *Dyak* in the *Dmel* species subgroup, using the newly sequenced genomes in the genus *Drosophila.* Although we found that tree 1, placing *Dere* and *Dyak* as sister species, is the best-supported tree, we found genome-wide incongruence in substitutions, insertions/deletions (indels), and gene trees. We show that the branch separating the split of *Dmel* from the split of *Dere* and *Dyak* is sufficiently short that incomplete lineage sorting is a plausible explanation for the incongruence. We further show that the support for the three possible trees is nonrandomly distributed across the genome such that adjacent genes supporting the same tree are more likely in regions of low recombination, and substitutions supporting the same tree are most enriched roughly on the same scale as estimates of linkage disequilibrium, consistent with theoretical predictions under the coalescent [49]. We tested for obvious systematic biases and found that no factor we examined could account for the incongruence. We conclude by suggesting that incongruence due to incomplete lineage sorting has important implications for comparative genomics research.

## Results

### Comparative Annotation of *Drosophila* Species

To analyze the phylogenetic history of the gene compliment of each of the seven fully sequenced species in the subgenus *Sophophora,* we mapped *Dmel* gene annotations onto each unannotated genome. *Dmel* coding sequences (19,186) were mapped to potential orthologous regions in each species using TBLASTN, and GeneWise was used to build gene models based on the *Dmel* gene in each region. These GeneWise models were matched back to *Dmel* translations using BLASTP, and genes for which clear orthologs could be found were used in downstream analysis (see Methods). Peptide sequences from orthologs were aligned using TCoffee [50] and cDNA alignments were mapped onto the peptide alignments.

### Species and Trees

Of these seven subgenus *Sophophora* species, we chose to use *Dmel, Dere, Dyak,* and *D. ananassae (Dana)* for our initial analysis of the placement of *Dere* and *Dyak* within the *Dmel* species subgroup (we examine the effects of species choice on our results below). *Dmel* was chosen because the annotations were mapped from *Dmel,* and it is the primary model organism of the subgenus. *D. simulans (Dsim)* and *D. sechellia (Dsec)* were excluded from initial analysis because they were assumed to provide mostly redundant information to *Dmel* and they reduced the number of clear orthologs spanning the species by 2,544 genes, presumably because of lower sequence coverage and issues regarding the assembly of polymorphic reads in *Dsim. Dana* was chosen over *D. pseudoobscura (Dpse)* because it is the closest fully sequenced outgroup to the *Dmel* species subgroup. More than 9,000 genes (9,405) were found to have clear orthologs in all four of the chosen species. Figure 1 shows the three possible unrooted trees relating the species.

### Genome-Wide Incongruence

**Figure 2:**
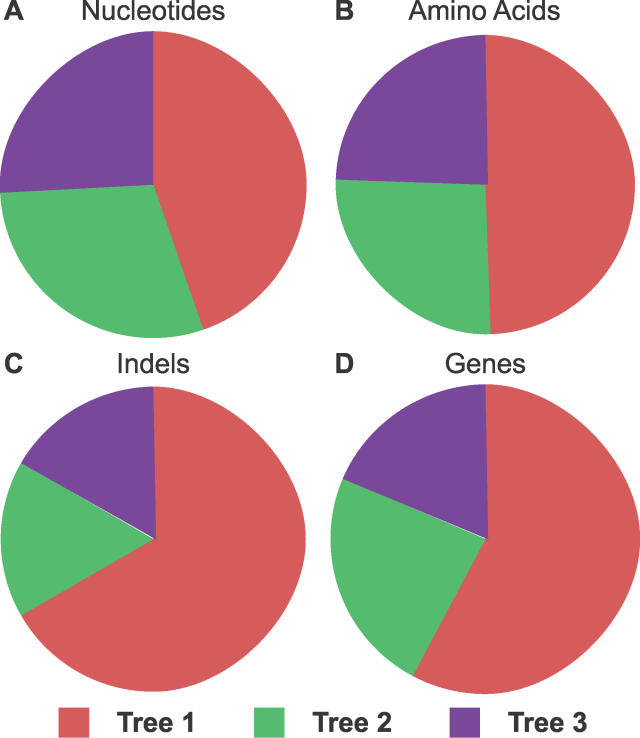
Widespread Incongruence of Substitutions, Indels, and Gene Trees (A) The proportion of informative nucleotide substitutions in 9,405 genes supporting each of the three trees. Tree 1 (red) is supported by 170,002 (44.7%) nucleotide changes; tree 2 (green), 112,278 (29.5%) nucleotide changes; and tree 3 (purple), 98,117 (25.8%) nucleotide changes. (B) The proportion of informative amino acid substitutions in 9,405 genes supporting each of the three trees. Tree 1 (red) is supported by 28,628 (49.3%) amino acid changes; tree 2 (green), 15,182 (26.2%) amino acid changes; and tree 3 (purple), 14,203 (24.5%) amino acid changes. (C) The proportion of informative insertions or deletions (indels) in 9,405 genes supporting each of the three genes. Indels were filtered, requiring five flanking amino acids of perfect identity and no repetitive sequence. Tree 1 (red) is supported by 2 deletions and 6 insertions (66.7%); tree 2 (green), 1 deletion and 1 insertion (16.7%); and tree 3 (purple), 2 insertions (16.7%). Similar proportions but much larger counts are found when the indels are not filtered. (D) The proportion of 9,315 genes with ML support for each of the three trees. Tree 1 (red) has ML support for 5,381 (57.8%); tree 2 (green), 2,188 (23.5%); and tree 3 (purple), 1,746 (18.7%).

We began our analysis looking directly at the genome-wide counts of amino acid substitutions, nucleotide substitutions, and indel events that were informative with respect to each of the three possible trees (see Methods). For all three characters, tree 1, which groups *Dere* and *Dyak* together, was found to have the most support (Figure 2A–2C). By a majority-rule consensus, tree 1 would be inferred to be the species tree, consistent with the findings of Ko et al. [8]. The high proportion of substitutions and indels supporting the alternate trees, however, suggests a poorly resolved tree and pervasive incongruence.

**Figure 3:**
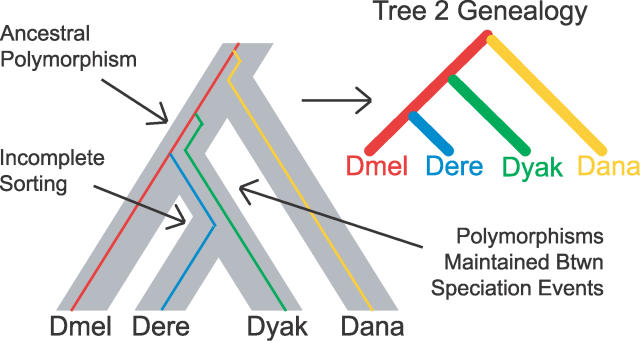
Incomplete Lineage Sorting The history of a gene (colored lines) is drawn in the context of a species tree (gray bars). New lineages arising from new polymorphisms in the gene are drawn in different colors. In this case, the two alleles in the population prior to the split of *Dmel* are maintained through to the split of *Dere* and *Dyak,* leading to incomplete lineage sorting and an incongruent genealogy (tree 2). The greater the diversity in the ancestral population and the shorter the time between speciation events, the more likely nonspecies genealogies are.

What is the cause of this incongruence? The incongruent substitutions could be the product of any of a number of systematic biases, but the incongruent indels are unambiguous characters that are more difficult to explain as methodological artifacts [51,52]. The population genetic theory of the coalescent states that sufficiently close speciation events will lead to incongruence due to incomplete lineage sorting (Figure 3) [38]. Below we explore the compatibility of our data with the coalescent as well as test for possible systematic biases.

### Maximum Likelihood Gene Trees Show Incongruence

We first repeated our analysis using maximum likelihood (ML) methods [53,54] to measure the informative divergence spanning the inferred speciation events and to test the robustness of the incongruent substitutions using more complex models of sequence evolution. ML analysis is not currently scalable to entire genomes in a single calculation, so we partitioned the genome into individual genes. If incomplete lineage sorting is the underlying cause of the incongruence, such a partition might also reveal variation in allelic histories that multigene concatenations could obscure [27,45,55]. Wanting to capture both the observed nucleotide and amino acid differences across the species [56], we used the F3×4 codon-based model from the PAML package [57] to compare the likelihood of each tree given each cDNA alignment (we test other models below). Consistent with the parsimony-based analysis, the majority of genes (57.8%) support tree 1, while a high proportion (42.2%) support the other two trees (Figure 2D).

**Figure 4:**
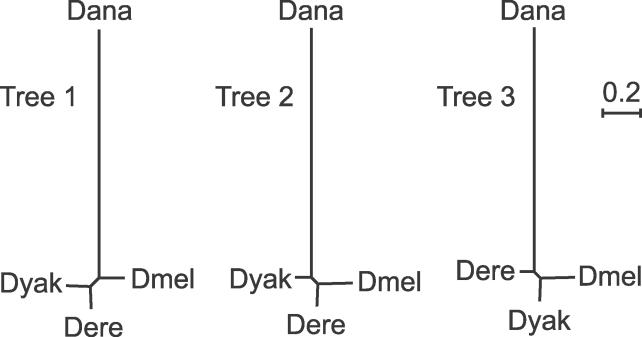
Median Synonymous Trees Median synonymous branch length trees derived from the genes supporting each of the three trees are drawn to the same scale. The branch spanning the two speciation events is quite short for all trees.

The median synonymous divergence trees for the sets of genes supporting each tree are: (dmel:0.1301,(dere:0.1095,dyak:0.1201):0.0664,dana:1.3246) for tree 1, ((dmel:0.1744,dere:0.1076):0.0498,dyak:0.0757,dana:1.2871) for tree 2, and ((dmel:0.1801,dyak:0.1163):0.0454,dere:0.0719,dana:1.3147) for tree 3 (Figure 4). The branches between the speciation events are quite short, with the tree 1 branch being the longest at only 0.066, suggesting that these species split in rapid succession.

### Incongruence Is Expected for These Species under the Coalescent

**Figure 5:**
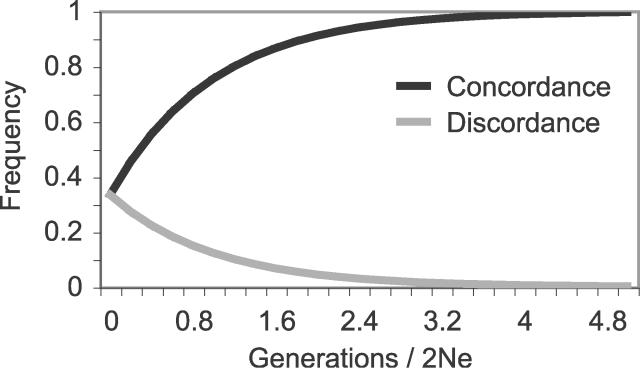
Coalescence Probabilities for Each Tree Using the formula p(congruence) = 1 − 2/3exp(−*t*), where *t* = generations / 2Ne, the probability of the species tree (black) and the probability of one of the two alternate trees (gray) was plotted as a function of *t*.

Is the time spanning these speciation events short enough to expect the observed levels of incongruence? Using the coalescent, the probability of congruence, or monophyly, can be directly calculated for the three-taxon case using the equation p(congruence) = 1 − 2 / 3exp(−*t*), where *t* is the time between speciation events in units of generations / 2Ne and *Ne* is the effective population size [58–60]. Figure 5 shows this probability graphically as a function of *t.* In order to go from an estimate of the informative divergence to this probability, the substitutions per site per year, the ancestral generation time and the ancestral population size must be known. Synonymous substitutions per site per year has been estimated to be in the range of 1–2 × 10^−8^ in *Drosophila* [1,13,61,62]. Generations per year for the extant taxa in the *Dmel* species subgroup is about ten and can be used as an estimate for the ancestral generation time [63]. The ancestral population size has been estimated in the range of 10^6^ to 10^7^, but this should be considered a poorly resolved parameter [64]. Theoretically, the median informative branch length measured above includes both divergence prior to the first speciation event and divergence between the two speciation events. If we take the informative divergence estimated from genes supporting the alternative trees to represent the expected amount of divergence prior to the first speciation event (0.05 and 0.045 for trees 2 and 3, respectively) and subtract their average (0.0475) from the tree 1 total informative divergence (0.066), we can get an estimate of the informative divergence spanning the two speciation events (0.019). This leads to an estimate of 9.5 × 10^5^ to 1.9 × 10^6^ years, or 9.5 × 10^6^ to 1.9 × 10^7^ generations. The range of values for *t* becomes 0.48 to 9.5, which produces probabilities for congruence in the range of 0.59 to 0.99995 (Figure 5). Although the uncertainty in these parameter estimates does not permit us to say that incongruence would be guaranteed, they do allow us to say that incongruence due to incomplete lineage sorting is expected under plausible assumptions about these species' ancestral population and speciation events.

### Spatial Structure of Tree Support

Given that we observed incongruence in individual sites as well as for whole genes, we wanted to better understand the extent to which sites supporting the same tree are spatially correlated, with a particular interest in the compatibility of this structure with the incomplete lineage-sorting hypothesis. The above analysis of gene trees suggests that sites can be correlated out to the length of genes. To see if this correlation extends beyond individual genes we looked for blocks of adjacent genes supporting the same gene and tested for unusual block lengths. Using permutations of ML gene tree states to obtain significance, we found gene tree block lengths at expected frequencies, with the exception of an excess of long blocks supporting tree 3 in the range of 250 kb to 700 kb, three of which were highly significant (*p* < 0.05).

If the blocks of genes supporting the same tree were the product of incomplete lineage sorting, then regions of low recombination ought to have larger blocks [65]. Although the ancestral recombination rates are not known, we looked to see if block lengths are correlated with *Dmel* recombination rates [66]. We found a weak negative correlation for all blocks (Pearson's R = −0.13, *p* < 0.1) as well for blocks for each specific tree, with tree 2 blocks showing the strongest correlation (Pearson's R = −0.30, *p* < 0.05). These weak correlations suggest a minor role for recombination rates in determining the spatial structure of support for different trees across the genome; however, there are many reasons for why strong correlations would not be expected, including poorly conserved recombination rates across these species [67–69] and gene conversion in regions of low recombination [70–72]. Nonetheless, these weak correlations establish a connection between recombination and the spatial structure of support that is at least consistent with lineage sorting. We next looked at the spatial correlation of individual sites to understand the spatial correlation at a finer scale.

**Figure 6:**
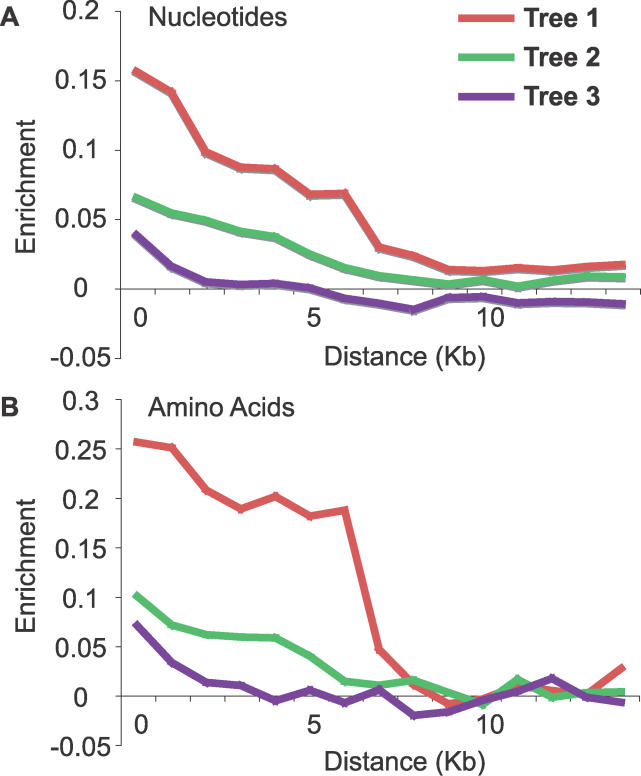
Clustering of Informative Sites The enrichment of informative nucleotide (A) and amino acid (B) substitutions near other substitutions that support the same phylogeny was found for all three trees and is on a scale roughly similar to estimates of linkage disequilibrium. At each informative site in the genome, the counts of informative sites supporting each of the three trees in 1-kb windows extending 30 kb up- and downstream were measured. For each type of informative site, the enrichment of the same type of informative site in each 1-kb window was calculated using the observed counts and the expected number of sites based on their genome-wide frequency. Enrichment is log_10_(observed / expected).

Using the whole-genome frequencies of informative amino acid and nucleotide substitutions supporting each tree, we looked to see if sites supporting the same tree are locally enriched across chromosomes (see Methods for more details). Figure 6 shows that informative amino acid and nucleotide substitutions supporting the same tree cluster together on the scale of less than 8 kb for trees 1 and 2 and less than 2 kb for tree 3. These local deviations in the frequencies of informative substitutions from the expected frequencies are quite highly significant (*X* ^2^ test, *p* < 10^−10^).

What forces might have shaped these clusters of informative sites supporting the same tree? Under the coalescent, linked neutrally evolving sites supporting the same tree have been proposed to be correlated at an expected distance equal to linkage disequilibrium [49]. Linkage disequilibrium in *Dmel* has been estimated to extend to the length of a few kilobases [73], suggesting that our results are consistent with theoretical expectations [49]. Theoretical considerations together with recent empirical evidence from *Dmel,* however, imply that neutral sites would not be expected to be in disequilibrium at distances greater than a few hundred base pairs [74,75], suggesting that perhaps selection has acted to increase the scale of these correlations [65]. Regardless of the influence of selection, the structure of the support for different trees across the genome is consistent with recombination acting within the context of incomplete lineage sorting.

Additional support for this conclusion comes from the observation that mitochondrial genes exhibit no incongruence (K. Montooth and D. Rand, personal communication). This is expected, as recombination is not thought to occur in the mitochondrial genome. While mitochondrial evolution differs from nuclear evolution in more ways than just recombination [76], the complete lack of incongruence is nevertheless striking.

Thus far we have presented results suggesting that incomplete lineage sorting is a plausible explanation for the observed incongruence. We next sought to rule out alternate explanations.

### Statistical Support for Incongruence

**Figure 7:**
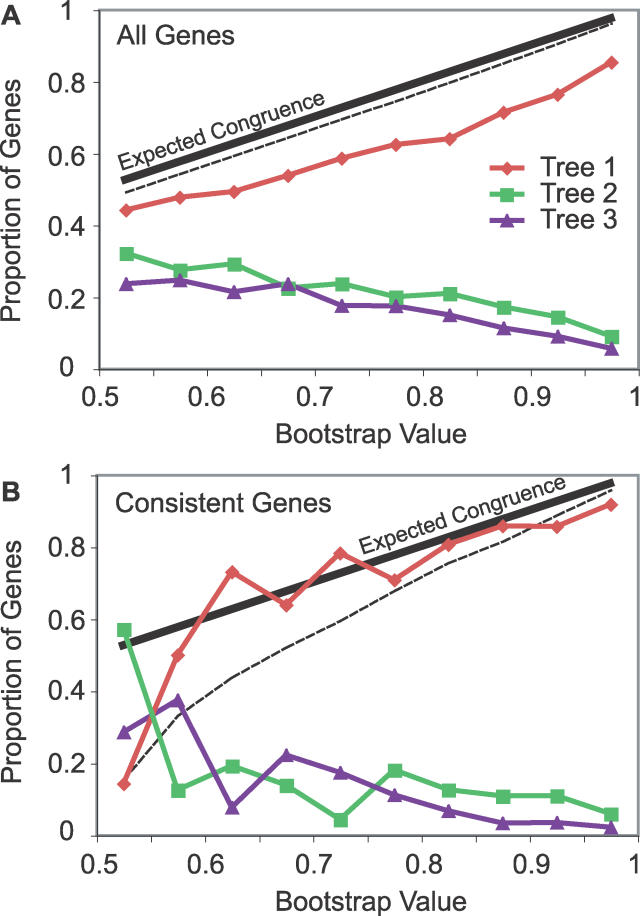
Significance of Incongruence An excess of incongruence above what is expected by chance was observed for the set of all genes (A) as well as the set of genes that consistently supported the same tree across models and species combinations (B). Genes were binned by bootstrap value, and the proportion of genes supporting tree 1 (red line), tree 2 (green line), and tree 3 (purple line) were plotted. The expected congruence based on the bootstrap value in each bin (black solid line) and the 95% confidence interval based on a *X* ^2^ distribution (black dash line) demonstrate the excess incongruence.

Is the incongruence in gene trees unexpected given the strength of support for each inference? To address this question, we used the bootstrap [77] value, RELL [78], from 10,000 replicates as an estimate of the expected incongruence due to chance alone. Taylor and Piel have shown that for a large set of yeast genes, originally reported by Rokas et al [79], there is no significant difference between nonparametric bootstrap values and accuracy, as measured by congruence [80]. Earlier work suggests that bootstrap values are conservative and likely to underestimate accuracy [81,82]. Figure 7A shows the proportion of genes supporting each tree in bins of bootstrap value. Unlike the yeast phylogeny, our observed incongruence consistently exceeds that expected by bootstrap values. Thus, the incongruence for these four species using the F3×4 codon model appears to be statistically significant.

### Incongruence Is Robust to Model Choice

We next tested whether the incongruence is robust to model choice. An empirical study of model choice and accuracy by Ren et al found that codon-based models are able to recover both recent and deep divergences well, while nucleotide-based models are less efficient at deep divergences and amino acid–based models are less efficient at recent divergences [56]. They also found that while more complex models fit the data better, they are not necessarily more accurate, a conclusion that has been made by other studies [83,84]. We looked at six models: nucleotide-based (HKY, HKY+G), codon-based (F3×4, F3×4+G), and amino acid–based (WAG+F, WAG+F+G) models both with and without a discrete gamma model of variable rates among sites (see Methods). Incongruence was found to exceed expected levels from bootstrap values across all models, suggesting that the incongruence is indeed robust to model choice (Figure S1).

Comparing congruence across models, simpler models seem to produce more congruence than more complex models (Table 1). For each of the three types of models, addition of a discrete gamma resulted in lower congruence. For the models without discrete gamma, HKY was more congruent than F3×4, which was more congruent than WAG+F, perhaps due to the relatively recent divergences in this phylogeny. Interestingly, the more complex models, F3×4+G for nucleotides and WAG+F+G for amino acids, fit the alignments better for most genes, according to Akaike's information criterion (Table 1) [85]. Thus, consistent with the finding of Ren et al. with the yeast dataset [56], more complex models fit the data better but produce less congruence.

**Table 1:**
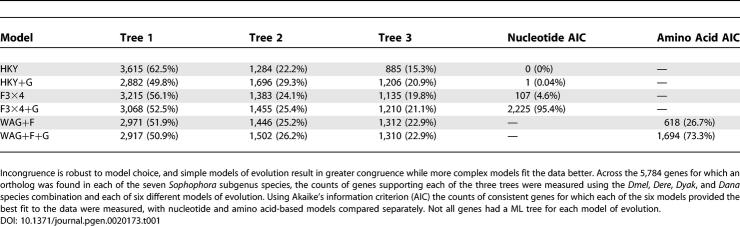
Congruence and Fit to Data across Six Models of Evolution

### Species Choice Does Not Explain the Observed Incongruence

To evaluate the robustness of the incongruence to species choice we examined the set of 5,784 genes for which a clear ortholog could be found in all seven fully sequenced species in the subgenous *Sophophora: Dmel, Dsim, Dsec, Dere, Dyak, Dana,* and *Dpse.* All 21 possible species combinations that include *Dere* and *Dyak* and at least one of *Dmel, Dsim,* and *Dsec,* as well as at least one of *Dana* and *Dpse,* were considered. The HKY model was used both because it was found to produce the most congruence in the original four species as well as because it is considerably more computationally efficient than the codon models. Across all species combinations, incongruence is consistently greater than expected from bootstrap values, suggesting that incongruence is not species choice dependent (Figures S1A and S2).

Ranking species combinations by levels of congruence reveals that our original species choice produces the most congruence (Table 2), suggesting that our estimates are conservative. The relative congruence of the species combinations appears nonrandom, with respect to presence or absence of individual species, so we calculated the average congruence for each species across the combinations containing that species. Although the average congruence is very similar for each species, we found that *Dana* (82.4%) contributes most to congruence, while *Dsim* (80.8%), *Dsec* (80.4%), and *Dpse* (79.7%) contribute roughly equally and *Dmel* (78.9%) actually contributes least to congruence. We note that the presence of *Dmel* in the most congruent species combination goes against this general trend, perhaps reflecting further complexities in the impact of species choice on congruence.

**Table 2:**
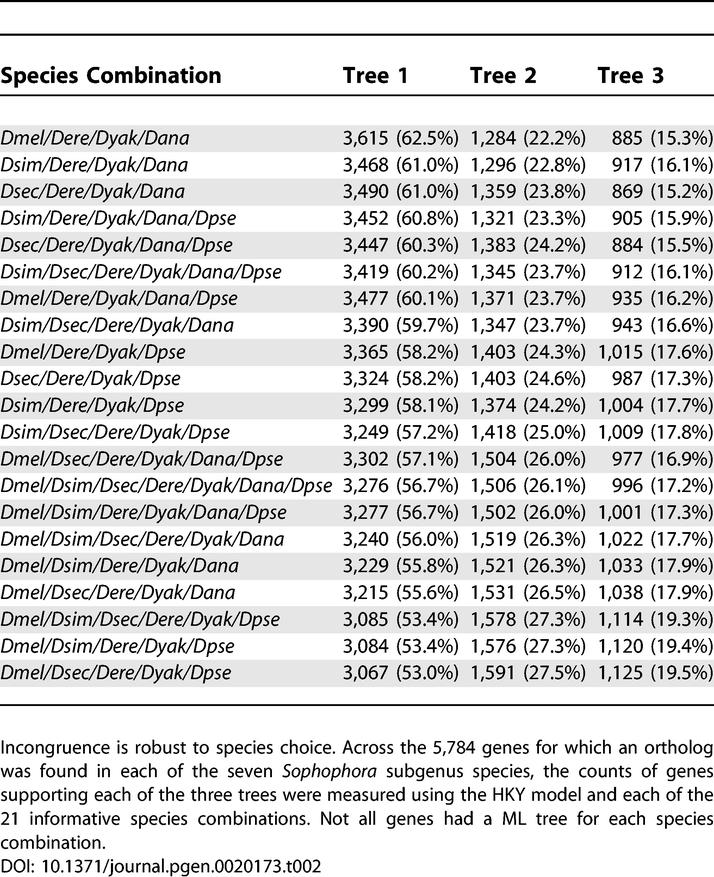
Congruence across 21 Species Combinations

### Consistency

Although the incongruence appears to be robust to model and species choice, a much more stringent test is to look at incongruence in the partition of genes that consistently support the same tree across all models and across all species combinations [86]. Of the 5,784 genes analyzed, 2,347 are consistent across all models and of those, 1,600 (68.2%) are congruent while 443 (18.9%) support tree 2 and 304 (12.9%) support tree 3. Similarly, 1,918 genes are consistent across species combinations and of those, 1,474 (76.8%) are congruent while 291 (15.2%) support tree 2 and 153 (8%) support tree 3. Finally, 970 genes are consistent across all models and all species combinations and of those, 804 (82.9%) are congruent, while 101 (10.4%) support tree 2 and 61 (6.3%) support tree 3. This conservative partitioning reduces the amount of incongruence but does not eliminate it. We note that under the incomplete lineage-sorting hypothesis, incongruent genes are expected to have accumulated fewer informative substitutions (Figure 4) and therefore might be expected to be less robust to such a consistency test.

To assess the statistical significance of the incongruence in the partition of genes consistent across all models and species combinations [31], we used the HKY model bootstrap values from the *Dmel, Dere, Dyak,* and *Dana* species combination to look at congruence as a function of bootstrap value. As shown in figure 7B, the congruence is less than expected for the highest bootstrap values. For the 521 genes with bootstrap values between 0.9 and 1.0, which is more than half of consistent genes, the incongruence was highly significant (*X* ^2^ test, *p* < 10^−3^).

To further test whether the statistical support from the incongruent genes is the result of consistent signal, as opposed to having hidden support [87] for tree 1, we concatenated the 804 consistent tree 1 genes, 101 consistent tree 2 genes, and 61 tree 3 genes into three large alignments and repeated the ML analysis for the *Dmel, Dere, Dyak,* and *Dana* species combination and the HKY model. Interestingly, each tree-specific concatenation supported its tree with 100% bootstrap support [88]. Thus, the signal for incongruence appears to be consistent, highly significant, and robust to model and species choice consistency partitioning.

### Sequence and Evolutionary Properties

**Figure 8:**
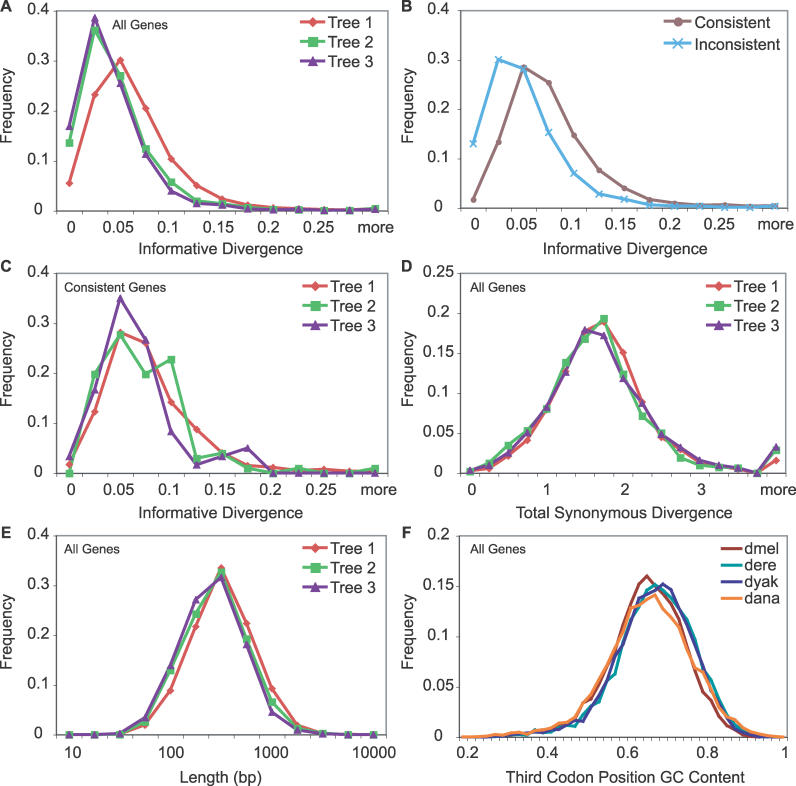
Sequence and Evolutionary Gene Properties Sequence and evolutionary properties of the genes are unable to explain the incongruence. Distributions are calculated using results from the original ML analysis using the F3×4 model and the *Dmel, Dere, Dyak,* and *Dana* species combination. The distributions of informative synonymous divergences in genes supporting each tree reveal a bias toward lower values for the incongruent genes (A). Nearly all genes with little or no informative synonymous divergence, however, are classified as inconsistent (B). Therefore, consistent genes have very similar distributions of ISD across trees (C). TSD is distributed similarly across trees, suggesting homoplasy due to increased mutation rates is not causing the incongruence (D). Gene length is slightly higher in tree 1 genes but overall is very similar across trees (E). Third codon position GC content is slightly biased toward lower values for *Dmel* and *Dana* and higher values for *Dere* and *Dyak,* creating a conservative bias for the incongruence (F).

We next looked at sequence and evolutionary properties of the genes supporting each tree to see if any clear biases could explain the incongruence. The properties we examined are sequence quality, gene length (measured in ungapped codons in the alignment), base composition (GC content) across the species at each position in the codon, transition–transversion ratio (kappa), ratio of nonsynonymous to synonymous divergence (dN/dS), informative synonymous divergence (ISD), ratio of informative synonymous divergence to noninformative synonymous divergence (RINSD), and total synonymous divergence (TSD). Table S1 shows the correlation of bootstrap values to each of these properties for the whole set of genes, genes supporting each tree, the set of genes found to be consistent across models and species combinations, the genes that consistently supported each tree, and the set of inconsistent genes. Distributions for each property are shown in Figures 8 and S3–S8.

The strongest and most consistent correlations with bootstrap value are for ISD and RINSD (Table S1), which are in essence the signal and signal to noise. We've already shown that the median informative divergence in the genes supporting tree 1 is greater than that for the genes supporting trees 2 and 3 (Figure 4). Reflecting this, the distributions of ISD and RINSD for genes supporting trees 2 and 3 are shifted toward lower values compared to genes supporting tree 1 (Figures 8A and S3A). Comparing consistent genes and inconsistent genes reveals that nearly all genes with ISD values close to zero are classified as inconsistent (Figure 8B). Among consistent genes, those supporting trees 2 and 3 still have distributions of ISD and RINSD shifted slightly toward lower values compared to those supporting tree 1 (Figures 8C and S3B). The fact that incongruent genes are expected to have lower ISDs than congruent genes under the incomplete lineage-sorting model (see above), and the fact the ISD and RINSD distributions are highly overlapping for each of the three trees, suggests that lack of signal or low signal to noise cannot explain the observed incongruence.

The long branch out to *Dana* (Figure 4) presents the concern that the incongruence may be due to homoplasy and perhaps long-branch attraction. TSD is distributed nearly identically across all sets of genes, including consistent and inconsistent genes, with a very slight bias toward trees 2 and 3 genes; inconsistent genes have lower TSDs (Figures 8D and S4). Although this does not rule out homoplasy as a source for noise in the inference of gene trees, it appears that regions with high mutational rates are not biased toward supporting incongruent or inconsistent genes [89], making it a less likely explanatory factor. In addition, although the trees in Figure 4 are not ultrametric (leaves equidistant from internal nodes), they are biased in the opposite direction as would be expected under long branch attraction, with the shortest branch in the *Dmel* species subgroup pairing with the longest branch out to *Dana.* Thus, homoplasy and long-branch attraction do not appear to be responsible for the incongruence.

Another possibility is that sampling variance in short genes is leading to the incongruence [90]. We've already shown that a concatenation of the consistent genes supporting each tree gives 100% bootstrap support, making sampling variance an unlikely explanation. Gene length is very similar across the sets of genes supporting each tree, but tree 1 genes tend to be slightly longer than genes supporting trees 2 and 3 (Figure 8E). Gene length is also weakly correlated with bootstrap value for the whole set, consistent genes, and tree 1 genes (both inconsistent and consistent) (Table S1). Our above results on the spatial correlation of sites, however, suggest that genes that extend more than a few kilobases would not be expected to be enriched for sites supporting the same tree above their background frequencies. We also found that enrichment is most pronounced for tree 1 sites and less so for incongruent sites. This increased mosaic structure [91] in incongruent genes is likely to be responsible for most of the shift to slightly larger genes in the tree 1 genes. The influence of sampling variance, however, is reflected in the shift of inconsistent genes compared to consistent genes toward shorter lengths. Thus, the small decrease in long genes in the incongruent set is probably a result of the spatial clustering of sites, while the small increase in short genes may be a combination of that effect and noise from sampling variance. Regardless, gene length is so similar across trees that it is unlikely to explain the incongruence.

GC content has been estimated to vary considerably across the species in the *Dmel* species subgroup [92] and is therefore a major concern for systematic bias. We found that GC content is highly similar across species at first and second codon positions, but varied systematically at the third codon position (Figures 8F, S5A, and S5B). *Dmel* and *Dana* have nearly identical distributions of third codon position GC content, which is shifted toward lower values compared to *Dere* and *Dyak,* which also have nearly identical distributions. This bias in GC content across species is very conservative with respect to the inference of incongruent genes because the incongruence would need to overcome the signal from base composition alone [29]. To further verify that this bias only works to decrease the incongruence, we converted the cDNA alignments into Rs and Ys, for purines and pyrimidines, respectively, and repeated the ML analysis using the F81 model of evolution, effectively averaging the contribution of GC and AT content and only measuring transversions [29,93,94]. As expected, incongruence actually increases (45.2%) under the RY coding and is still statistically significant (Figure S9). Other methods, for example those of Galtier and Gouy [95,96] and Gu and Li [97], attempt to explicitly model nonstationary evolution, rather than control for it. These methods might reveal more precisely the underestimation of incongruence due to the base composition bias in these species but are not expected to provide an explanation for the observed incongruence.

Sequence qualities, transition–transversion ratios, and dN/dS values were found be distributed similarly across trees, suggesting they are unlikely factors for systematic bias (Figures S6–S8).

### Sequence Properties Associated with Spatial Clustering

We last looked to see if the spatial clustering of sites supporting the same tree could be explained by evolutionary rate or base composition variation. To examine the relationship of evolutionary rate and the clustering of sites supporting each tree, we measured total divergence and the fraction of sites supporting each tree in overlapping windows across the chromosomes. For windows of sizes 5 kb or 1 kb, no correlation could be found between divergence and the fraction of sites supporting each tree, suggesting that evolutionary rate is unlikely to explain the spatial clustering. To test whether changes in GC content could explain the clustering of sites we used the RY-coded alignments (described above) [29,93,94] and repeated the spatial clustering analysis. Figure S10 shows that sites are still correlated in a similar range of a few kilobases, suggesting that variance in GC content is unlikely to be causing the spatial clustering of sites. Thus, both the incongruence as well as the spatial clustering of sites appear to be robust to the sequence and evolutionary properties examined.

## Discussion

We initially set out to confirm the placement of *Dere* and *Dyak* as sister species, relative to the *Dmel* lineage, in the *Dmel* species subgroup, using the fully sequenced genomes of seven species in the subgenus *Sophophora.* Although we did find that the best-supported phylogeny is that which places *Dere* and *Dyak* as sister species, we also found pervasive incongruence of substitutions, indels, and gene trees (figure 2). While incongruence in substitutions and gene trees could be the result of systematic biases, the incongruent indels, particularly unique insertions, presented strong enough evidence for unbiased incongruence that we also considered incomplete lineage sorting as a possible explanation. Assuming plausible values of substitution rate, generation time, and ancestral population size, we found that the time between the split of *Dmel* and the split of *Dere* and *Dyak* is sufficiently short that incomplete lineage sorting would be expected (Figures 3–5). Interestingly, we observed that the support for each of the three trees has a spatial structure across the genome, which is related to low recombination, both locally and globally (Figure 6). This further supports the hypothesis that the observed incongruence is due, at least in part, to incomplete lineage sorting.

To test for other plausible explanations we examined model choice, species choice, and variation in sequence and evolutionary properties and found no obvious candidate factors to explain the incongruence or the spatial structure of support for trees (Tables 1 and 2; Figures 7, 8, S1–S10). We therefore conclude that incomplete lineage sorting is the best-going explanation for the lack of resolution in this phylogeny.

Nevertheless, we likely did not exhaust the possible tests for alternate hypotheses for incongruence and suspect that this dataset will prove an interesting area for systematic research, much as the Rokas et al. yeast dataset has [69]. Comparing our results to the yeast dataset reveals important differences: there is significant incongruence beyond what would be expected by chance (Figure 7A), the level of incongruence is relatively robust to model choice (Tables 1 and 2; Figures 7B and S1), and basic sequence properties, like GC content, vary in ways that are conservative with respect to the incongruence (Figures 8, S3–S10) [29]. Similar to the yeast dataset, however, we find that the evolutionary model that maximizes the congruence (or accuracy, as Ren et al. refer to it) is typically the simplest (HKY), while the model that fits the data best is the most complex (F3×4 +G) (Table 1) [56].

To further understand the extent and nature of incomplete lineage sorting in the *Dmel* species subgroup, we suggest several types of future studies. First, to further test the agreement of the observed incongruence with theoretical predictions, better estimates of the ancestral effective population size, mutation rates, time between speciation events, ancestral recombination events [98], and examining the effects of selection (both directional and balancing [99]) would be of clear benefit. In addition, of great interest will be studies of lineage sorting across all taxa in the species group (especially the *Dsim* species complex [39]) and the influence of migration and gene flow on the symmetry of lineage sorting (because tree 2 is asymmetrically favored). Genome-wide population data already exist for *Dsim* and are expected for *Dmel,* which have the potential to help in the effort to understand these processes. Finally, methodological improvements might include increased large-scale taxon sampling, particularly from closely related taxa outside the species subgroup, such as the D. suzukii and D. takahashii subgroups [3], would alleviate potential biases introduced by the long branches out to *Dana* and *Dpse.*

Although this study should prove quite valuable to the increasing numbers of comparative genomics researchers studying the genus *Drosophila,* we believe our findings have important implications for comparative genomics as a whole. The idea that speciation events have occurred in rapid bursts throughout the tree of life [100–102] is likely broadly understood (for example, the short branch connecting the human, mouse, and dog lineages [103]), but the idea that genomes may be mosaics of conflicting genealogies as a result of rapid speciation is perhaps less well appreciated. As more species are sequenced, particularly the dense taxon sampling that is currently beginning in model organism clades, increasing numbers of close speciation events will likely result in many cases of incomplete lineage sorting in genome-scale data. As many methods used in comparative genomics require an accurate phylogeny, the comparative genomics community must develop methods that are robust to or take into account variation in phylogeny.

We envision three types of methods that will need to be developed to appropriately account for this kind of variation. The first are methods that can infer the most likely species tree using an entire genome in a single calculation, considering lineage sorting explicitly. The second are methods that can infer the most likely history of every base in every species, given the species tree. Last, comparative genomics methods that use phylogenies would need to be altered to control for and utilize the output from the second kind of method. Progress is being made in the first two categories [27,38,47,48,98,104–113], although no currently available method can deal with a whole-genome dataset such as this one. Though well appreciated in the systematics and population genetics communities, the issue of incomplete lineage sorting is rarely considered in the bioinformatics and comparative genomics communities, so the third category of method is virtually nonexistent. Accounting for variation in evolutionary histories will have different effects on different classes of methods, but we suggest that parsimony-based methods would be most strongly affected. An important example of such a phylogeny-based method is genome-wide multiple alignment using a guide tree (i.e., [114,115]), which is the first step in nearly all comparative genomic analyses. The availability of genome-scale datasets such as the one analyzed here should allow rapid progress in all three of these types of methods; we suggest that their development will be of great benefit to the evolutionary and comparative genomics community in the near future.

## Materials and Methods

### Assemblies.

*Dmel* release 4.2 genome, cDNA, and translation sequences were downloaded from Flybase (http://www.flybase.net). Prepublication assemblies for *Dere* and *Dana* (dated August 1, 2005), sequenced and assembled by Agencourt Bioscience (http://www.agencourt.com), and for *Dsec* (dated October 28, 2005), assembled and sequenced by the Broad Institute (http://www.broad.mit.edu), were downloaded from the Berkeley AAA website (http://rana.lbl.gov/drosophila). The prepublication assemblies for *Dyak* (dated July 4, 2004) and *Dsim* (dated June 2, 2005) were downloaded from the Washington University School of Medicine Genome Sequencing Center's website (ftp://genome.wustl.edu/pub). The *Dpse* v1.04 assembly was downloaded from Flybase. Assemblies can be found in Datasets S1–S6. Sequencing traces corresponding to these genomes are in the National Center for Biotechnology Information (NCBI) trace archive (http://ncbi.nlm.nih.gov/Traces/trace.cgi; *species_code,* “DROSOPHILA ERECTA,” “DROSOPHILA YAKUBA,” “DROSOPHILA ANANASSAE,” “DROSOPHILA SIMULANS,” “DROSOPHILA SECHELLIA,” “DROSOPHILA PSEUDOOBSCURA”).

### Comparative annotation.

Each of the sequence assemblies were annotated separately by mapping *Dmel* gene models onto the unannotated genome in a pairwise fashion using a modified reciprocal–BLAST approach [116] to assign orthology/paralogy relationships, and a comparative gene finder, GeneWise [117,118], to build gene models. The annotation pipeline consisted of three steps. (1) For each *Dmel* translation, we used the protein sequence as a NCBI TBLASTN [119] query (e-value threshold, 1 × 10^−^3) against the scaffolds of the target assembly. (2) The scaffolds were ordered by the hit e-value reported by TBLASTN, and up to two regions were selected from the two best scaffolds and used as input to construct gene models using GeneWise. To improve the chance of constructing a complete gene model using GeneWise, the regions were selected by clustering high-scoring pairs on the scaffold such that every high-scoring pair within 100 kb of another high-scoring pair was included in the same region, and a buffer of 10 kb was included at the ends of the regions. (3) The predicted translations of the models reported by GeneWise were then used as BLASTP queries against a database of *Dmel* translations, with an e-value threshold of 1 × 10^−^3.

We then assigned orthology/paralogy relationships using a heuristic algorithm that takes into account (1) the rank of the starting *Dmel* translation in the BLASTP results, (2) the rank of alternative translations from the gene corresponding to the starting *Dmel* translation, and (3) whether or not there were highly ranked hits to genes other than the gene corresponding to the starting *Dmel* translation. One-to-one orthology was assigned when the only top-ranked hits in the BLASTP results were translations from the gene corresponding to the starting *Dmel* translation. Hits that had e-values within one order of magnitude were considered equivalently ranked. For genes with more than one translation with clear orthologs in each species, the first historically annotated (translation with the lowest letter ID) was used to represent the gene.

cDNA and translation sequences can be found in Datasets S7–S18.

### Informative substitutions and indels.

Informative substitutions supporting each tree were counted across all cDNA and peptide alignments. Only single substitutions that split the four species into two groups of two were considered. Informative substitutions for tree 1 grouped *Dmel* and *Dana* together and *Dere* and *Dyak* together. Likewise, tree 2 grouped *Dmel* and *Dere* together and tree 3 grouped *Dmel* and *Dyak* together.

Informative indels supporting each tree were counted across all peptide alignments. Indels were classified as informative in the same way that substitutions were. Indels were further filtered to avoid artifacts from alignment errors. Only indels with five amino acids of perfect identity in flanking sequences, with no mono-, di-, or tri-amino acid repeats, were included. Insertions were inferred based on an absence in *Dana* and one of the ingroup species. Such insertions, where the inserted sequence is the same in the two species containing it, provided strong, unambiguous characters.

### ML gene trees.

The Codeml program of the PAML package (version 3.14) [57,120] was run on each gene using the following three unrooted trees: tree 1, *((Dmel,(Dere,Dyak),Dana);* tree 2, *((Dmel,Dere),Dyak,Dana);* and tree 3, ((*Dmel*,*Dyak*),*Dere*,*Dana*) (see Figure 1). Codeml was run using the F3×4 model, such that equilibrium codon frequencies were calculated from the average nucleotide frequencies at the three codon positions (CodonFreq = 2), amino amino acid distances were equal (aaDist = 0), one dN/dS value was estimated for all lineages using an initial value of 0.4 (model = 0, fix_omega = 0, omega = 0.4), the transition–transversion ratio was estimated with an initial value of 2 (fix_kappa = 0, kappa = 2), substitution rates across sites were set to be equal (fix_alpha = 1, alpha = 0), substitution rates were allowed to vary freely across lineages (clock = 0), and codons with ambiguous positions (gaps or Ns) were ignored (cleandata = 1).

### Spatial analysis.

Based on the ML tree for each gene, the genome was divided up into blocks supporting each tree. A ten-gene sliding window was used to calculate a running average of the support for each tree along each chromosome. Each window was assigned a tree based on the most frequent genealogy in the window. Each gene was then reassigned a tree based on the most frequent tree of all the windows that contained it. This effectively allows the neighbors of a gene to influence its assignment, and near neighbors have more influence than far neighbors. Adjacent genes that support the same tree were combined together into blocks. To measure the significance of the size of the blocks, the labels for each gene in the genome were randomized 1,000 times and the blocks were recalculated for each replicate, using the windowing method described above. Recombination rates for a subset of genes in *Dmel,* calculated by Hey and Kliman [66] using the *R* statistic, were downloaded. The average *R* in each block was calculated where a gene could be found in their set. The Pearson correlation of the average *R* within blocks and the length of blocks was calculated using the R statistics package [121].

Informative substitutions in genes were used to look at the structure of support for the different trees across the genome independent of the likelihood inference. The counts of each type of informative substitution were calculated in 60 nonoverlapping 1-kb windows surrounding each informative substitution across all chromosomes. The frequency of each kind of informative substitution across the whole genome was used to calculate an expected count for each 1-kb window. In each window, the enrichment of informative substitutions supporting the same tree was calculated. The *X* ^2^ significance of windows was calculated by comparing the observed frequencies of informative mutations supporting each tree with the genome averages of those frequencies.

### Bootstrap values.

RELL bootstrap values [78] from 10,000 replicates were taken from the Codeml output.

### PAML models.

All models were run using the same settings as described above for F3×4 except where HKY (model = 4) or WAG+F (model = 3) was specified and where the gamma function was used (fix_alpha = 0, alpha = 1.0, ncatg = 8).

### Akaike's information criterion.

Akaike's information criterion (AIC) was calculated as AIC = −2 ln L + 2 N, where *L* is the likelihood of the model given the data, and *N* is the degrees of freedom [85]. Only consistent genes were used in this analysis, so the tree was the same across all models. The likelihood and degrees of freedom were taken directly from PAML output. HKY, HKY+G, F3×4, and F3×4+G were compared, and WAG+F and WAG+F+G were compared.

### Sequence and evolutionary properties analysis.

The sequence quality in each species was calculated as the mean sequence quality score of the coding bases. Bootstrap value, length, GC content, transition–transversion ratio, dN/dS, ISD, NSD, and TSD were taken directly from the PAML output for the ML tree from the original analysis using the F3×4 model and the *Dmel*, *Dere*, *Dyak,* and *Dana* species combination. The Spearman rank correlations were calculated using the R statistics package [121].

### Divergence windows.

To examine the correlation of divergence with the proportion of sites supporting each tree in local areas across the genome we used 5-kb and 1-kb windows, overlapping by 2.5 kb and 0.5 kb, respectively. Using the synonymous site divergences reported by Codeml from the original analysis, we calculated the synonymous divergence per coding site in each window. We also calculated the proportion of sites supporting each tree in each window. Windows with no synonymous coding sites were excluded.

## Supporting Information

Dataset S1 Whole-Genome Assembly of *Dsim* in Fasta Format (38 MB ZIP)

Dataset S2 Whole-Genome Assembly of *Dere* in Fasta Format (42 MB ZIP)

Dataset S3 Whole-Genome Assembly of *Dsec* in Fasta Format (45 MB ZIP)

Dataset S4 Whole-Genome Assembly of *Dyak* in Fasta Format (51 MB ZIP)

Dataset S5 Whole-Genome Assembly of *Dpse* in Fasta Format (41 MB ZIP)

Dataset S6 Whole-Genome Assembly of *Dana* in Fasta Format (61 MB ZIP)

Dataset S7 cDNA Sequences for the Set of Clear Orthologs Annotated in *Dsim* in Fasta Format (4.2 MB ZIP)

Dataset S8 Peptide Sequences for the Set of Clear Orthologs Annotated in *Dsim* in Fasta Format (2.7 MB ZIP)

Dataset S9 cDNA Sequences for the Set of Clear Orthologs Annotated in *Dsec* in Fasta Format (4.7 MB ZIP)

Dataset S10 Peptide Sequences for the Set of Clear Orthologs Annotated in *Dsec* in Fasta Format (3.0 MB ZIP)

Dataset S11 cDNA Sequences for the Set of Clear Orthologs Annotated in *Dere* in Fasta Format (6.3 MB ZIP)

Dataset S12 Peptide Sequences for the Set of Clear Orthologs Annotated in *Dere* in Fasta Forma (4.0 MB ZIP)

Dataset S13 cDNA Sequences for the Set of Clear Orthologs Annotated in *Dyak* in Fasta Format (5.5 MB ZIP)

Dataset S14 Peptide Sequences for the Set of Clear Orthologs Annotated in *Dyak* in Fasta Format (3.5 MB ZIP)

Dataset S15 cDNA Sequences for the Set of Clear Orthologs Annotated in *Dana* in Fasta Format (5.8 MB ZIP)

Dataset S16 Peptide Sequences for the Set of Clear Orthologs Annotated in *Dana* in Fasta Format (3.8 MB ZIP)

Dataset S17 cDNA Sequences for the Set of Clear Orthologs Annotated in *Dpse* in Fasta Format (5.5 MB ZIP)

Dataset S18 Peptide Sequences for the Set of Clear Orthologs Annotated in *Dpse* in Fasta Format (3.5 MB ZIP)

Figure S1 Significance of Incongruence under Six Evolutionary Models An excess of incongruence above what is expected by chance was observed for genes from *Dmel, Dere, Dyak,* and *Dana* using the HKY model (A), the HKY+G model (B), the F3×4 model (C), the F3×4+G model (D), the WAG+F model (E), and the WAG+F+G model (F). Genes were binned by bootstrap value, and the proportion of genes supporting tree 1 (red line), tree 2 (green line), and tree 3 (purple line) were plotted. The expected congruence based on the bootstrap value in each bin (black solid line) demonstrates the excess incongruence.
(829 KB EPS)

Figure S2 Significance of Incongruence for 20 Species Combinations An excess of incongruence above what is expected by chance was observed using the HKY model for genes from *Dmel, Dsec, Dsim, Dere, Dyak, Dana,* and *Dpse* (A), *Dmel, Dsec, Dsim, Dere, Dyak,* and *Dana* (B), *Dmel, Dsec, Dsim, Dere, Dyak,* and *Dpse* (C), *Dmel, Dsec, Dere, Dyak, Dana,* and *Dpse* (D), *Dmel, Dsim, Dere, Dyak, Dana,* and *Dpse* (E)*, Dsec, Dsim, Dere, Dyak, Dana,* and *Dpse* (F), *Dsec, Dere, Dyak, Dana,* and *Dpse* (G), *Dmel, Dsim, Dere, Dyak,* and *Dana* (H), *Dsim, Dere, Dyak, Dana,* and *Dpse* (I), *Dmel, Dsec, Dere, Dyak* and *Dana* (J), *Dsim, Dere, Dyak,* and *Dana* (K), *Dsec, Dsim, Dere, Dyak,* and *Dana* (L), *Dsec, Dere, Dyak,* and *Dana* (M), *Dmel, Dsec, Dere, Dyak,* and *Dpse* (N), *Dmel, Dsim, Dere, Dyak,* and *Dpse* (O), *Dmel, Dere, Dyak,* and *Dpse* (P), *Dsec, Dsim, Dere, Dyak,* and *Dpse* (Q), *Dsim, Dere, Dyak,* and *Dpse* (R), *Dsec, Dere, Dyak,* and *Dpse* (S), and *Dmel, Dere, Dyak, Dana,* and *Dpse* (T). Genes were binned by bootstrap value, and the proportion of genes supporting tree 1 (red line), tree 2 (green line), and tree 3 (purple line) were plotted. The expected congruence based on the bootstrap value in each bin (black solid line) demonstrates the excess incongruence.
(68 KB PDF)

Figure S3 RINSD Although the distributions of the RINSD for incongruent genes are biased toward lower values relative to congruent genes for the set of all genes (A), distributions are similar across trees for the set of consistent genes. Distributions were calculated using results from the original ML analysis using the F3×4 model and the *Dmel, Dere, Dyak,* and *Dana* species combination.
(719 KB EPS)

Figure S4 TSD TSD is distributed similarly across consistent and inconsistent genes (A) as well as across trees for consistent genes (B), with a slight bias toward lower values for inconsistent genes and consistent genes supporting trees 2 and 3. Distributions were calculated using results from the original ML analysis using the F3×4 model and the *Dmel, Dere, Dyak,* and *Dana* species combination.
(701 KB EPS)

Figure S5 First and Second Codon Position GC Content GC content is distributed nearly identically across species for first (A) and second (B) codon positions in all genes. Distributions were calculated using results from the original ML analysis using the F3×4 model and the *Dmel, Dere, Dyak,* and *Dana* species combination.
(677 KB EPS)

Figure S6 Sequencing Quality Scores Mean sequencing quality scores for coding nucleotides in a gene are distributed nearly identically across trees in the set of all genes for *Dere* (A), *Dyak* (B) and *Dana* (C). Distributions were calculated using results from the original ML analysis using the F3×4 model and the *Dmel, Dere, Dyak,* and *Dana* species combination.
(845 KB EPS)

Figure S7 Transition–Transversion Ratio Transition–transversion ratios are similarly distributed across trees for the set of all genes. Distributions were calculated using results from the original ML analysis using the F3×4 model and the *Dmel, Dere, Dyak,* and *Dana* species combination.
(657 KB EPS)

Figure S8 dN/dS dN/dS values are similarly distributed across trees for the set of all genes. Distributions were calculated using results from the original ML analysis using the F3×4 model and the *Dmel, Dere, Dyak,* and *Dana* species combination.
(657 KB EPS)

Figure S9 Significance of Incongruence under RY Coding and F81 Model An excess of incongruence above what is expected by chance was observed for genes from *Dmel, Dere, Dyak,* and *Dana* using RY coding and the F81 model. Genes were binned by bootstrap value, and the proportion of genes supporting tree 1 (red), tree 2 (green line), and tree 3 (purple line) were plotted. The expected congruence based on the bootstrap value in each bin (black solid line) demonstrates the excess incongruence.
(644 KB EPS)

Figure S10 Clustering of Informative Sites with RY Coding Controlling for differences in GC content using RY coding, the enrichment of informative nucleotide substitutions near other substitutions that support the same phylogeny was found for all three trees and is on a scale roughly similar to estimates of linkage disequilibrium. At each informative site in the genome, the counts of informative sites supporting each of the three trees in 1-kb windows extending 30 kb up- and downstream were measured. For each type of informative site, the enrichment of the same type of informative site in each 1-kb window was calculated using the observed counts and the expected number of sites based on their genome-wide frequency. Enrichment is log_10_ (observed / expected).
(645 KB EPS)

Table S1 Spearman Rank Correlations of Sequence and Evolutionary Properties with Bootstrap Values across Sets of Genes (26 KB XLS)

## Abbreviations

*Dana,*: Drosophila ananassae;
*Dere,*: D. erecta;
*Dmel*: D. melanogaster;
dN/dS: ratio of nonsynonymous to synonymous divergence
*Dpse,*: D. pseudoobscura
*Dsec,*: D. sechellia;
*Dsim,*: D. simulans;
*Dyak,*: D. yakuba;
indel: insertion/deletion
ISD: informative synonymous divergence
ML: maximum likelihood
RINSD: ratio of informative synonymous divergence to non-informative synonymous divergence
TSD: total synonymous divergence
