# Supplementary figures and images for: Widespread Discordance of Gene Trees with Species Tree in Drosophila: Evidence for Incomplete Lineage Sorting
Source: PLoS Genet. 2006 Oct 27;2(10):e173. doi: 10.1371/journal.pgen.0020173 (PMC1626107; doi:10.1371/journal.pgen.0020173)

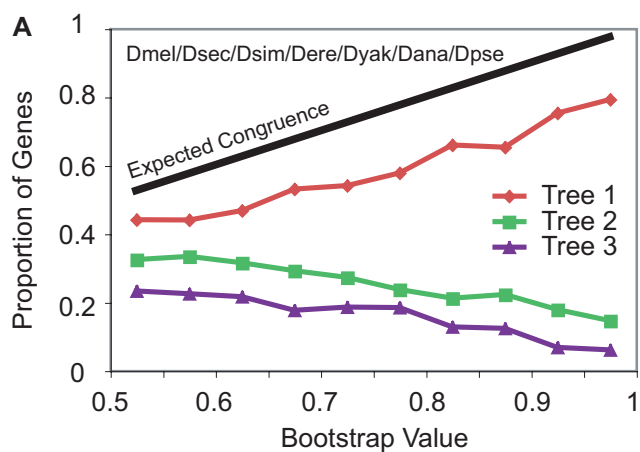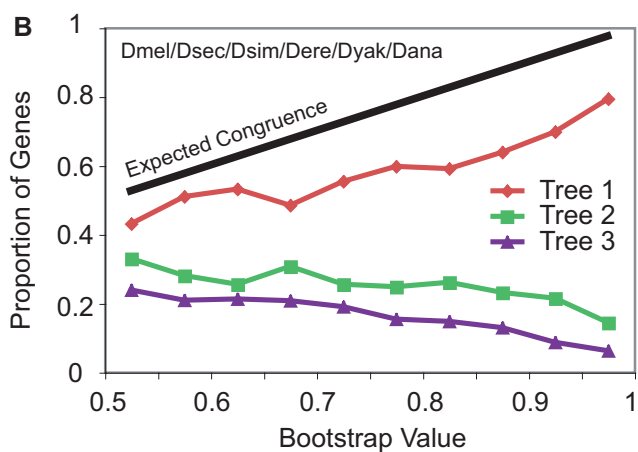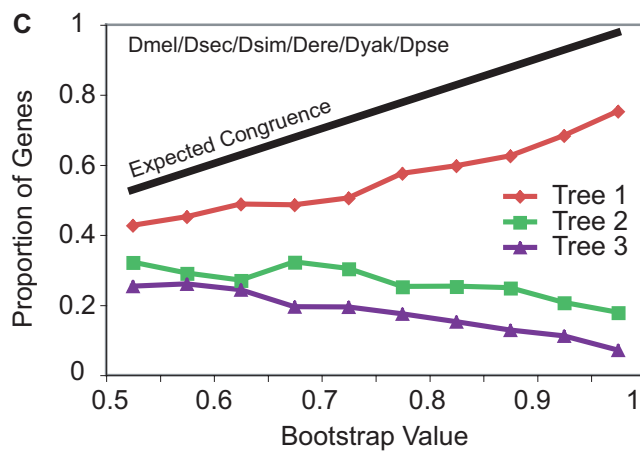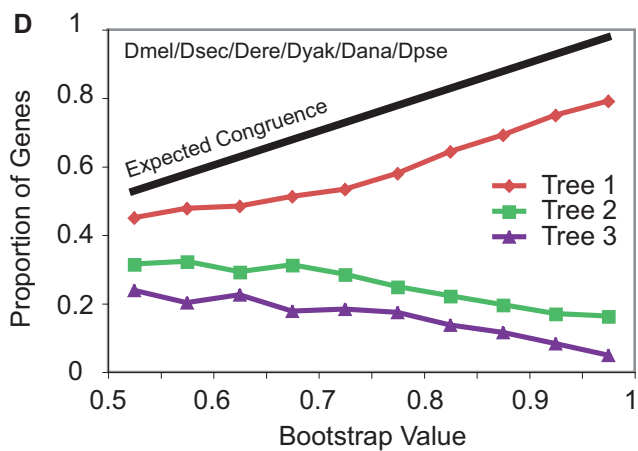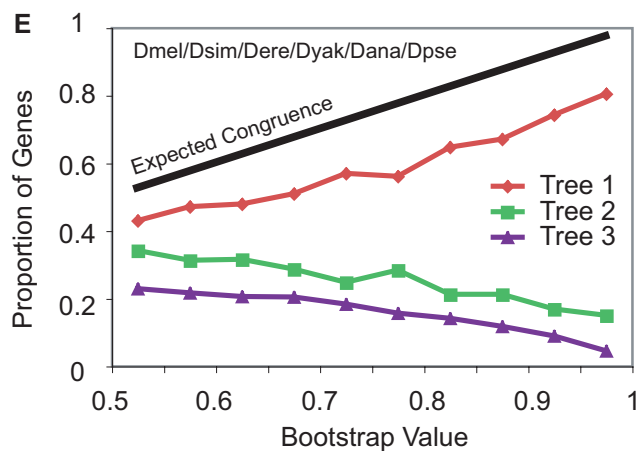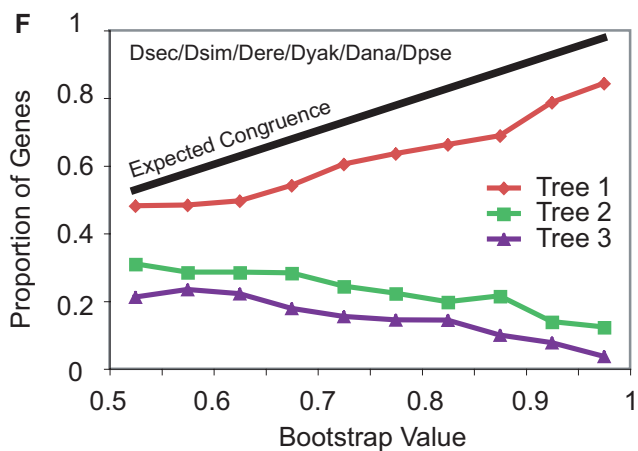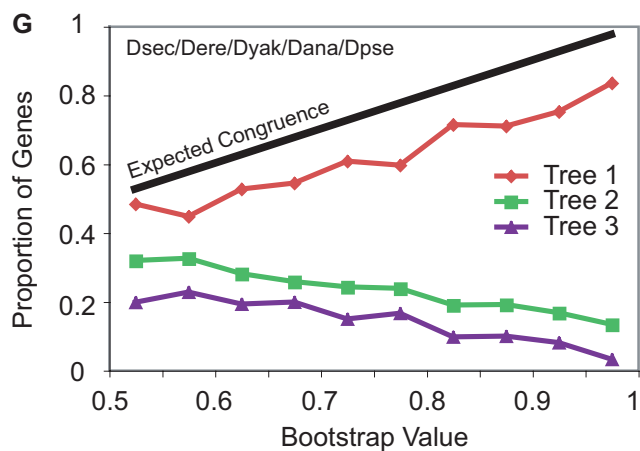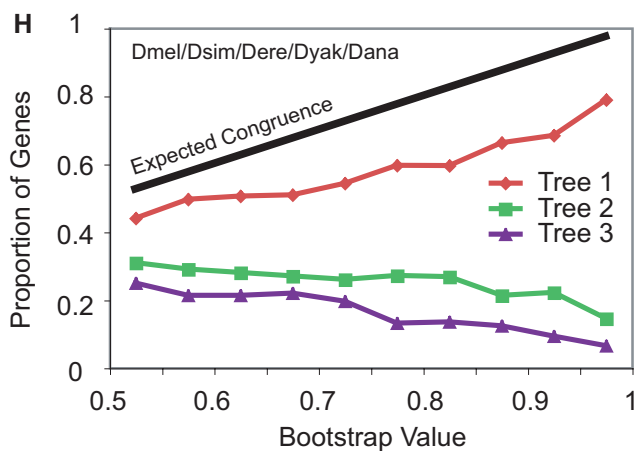

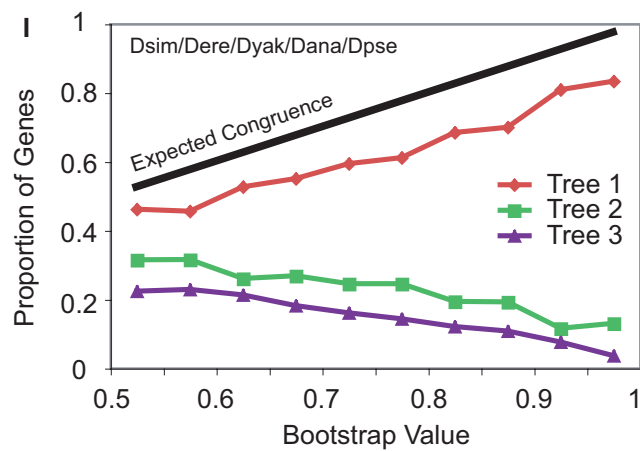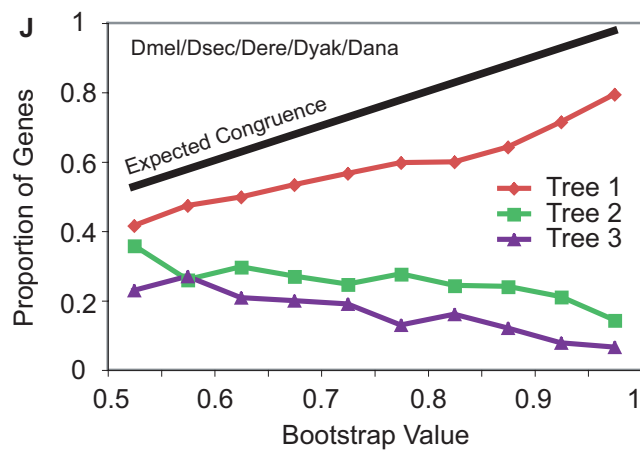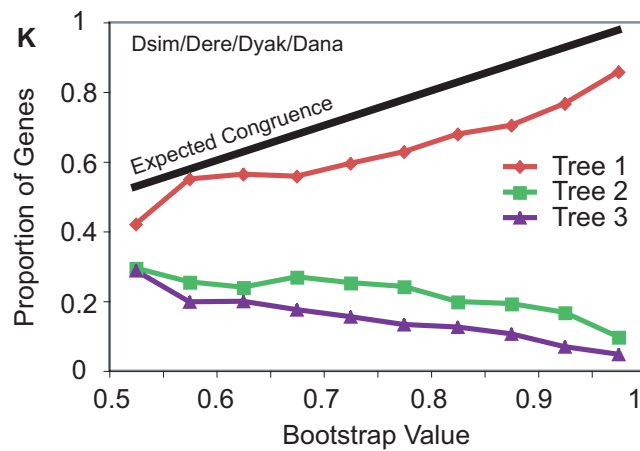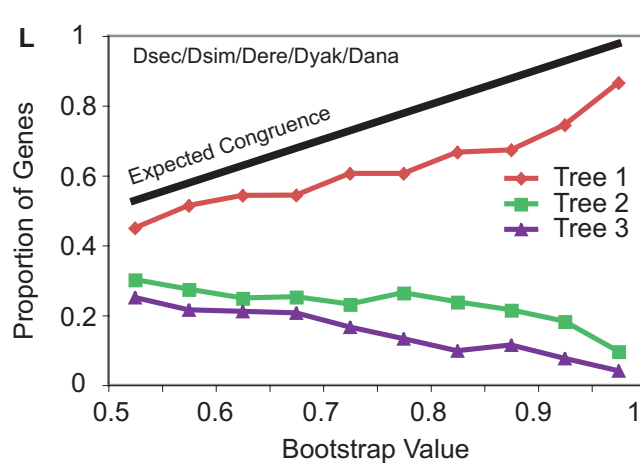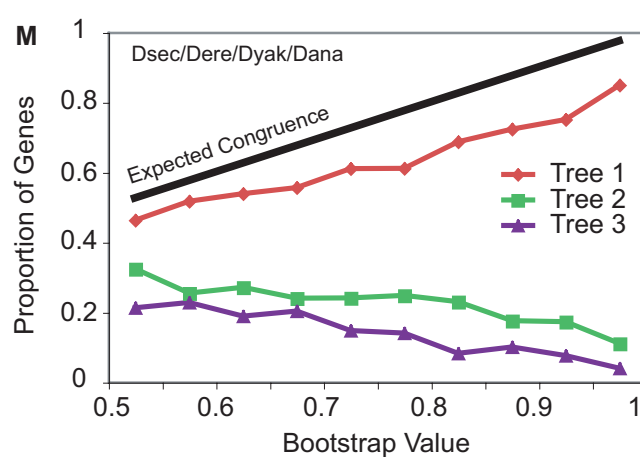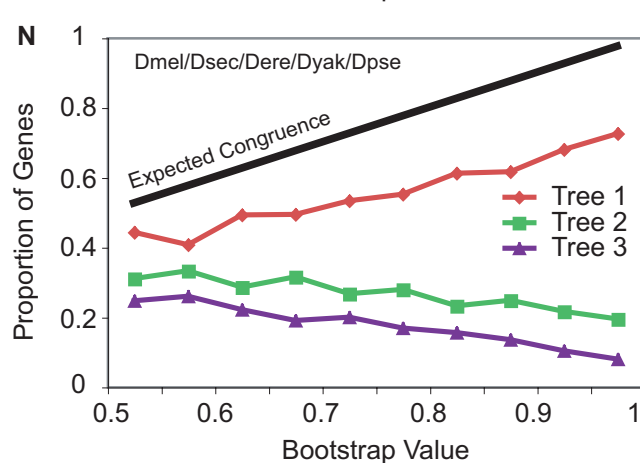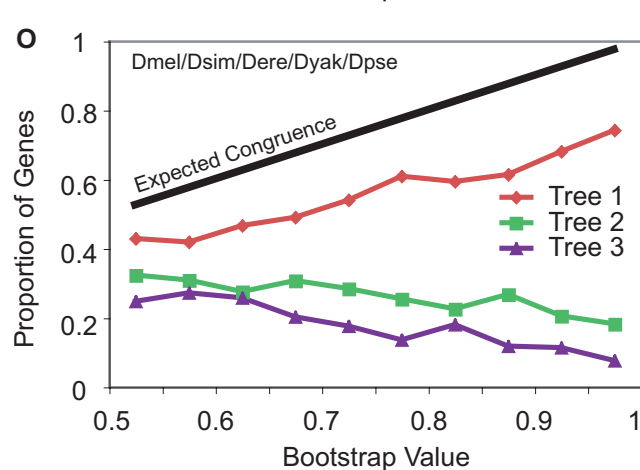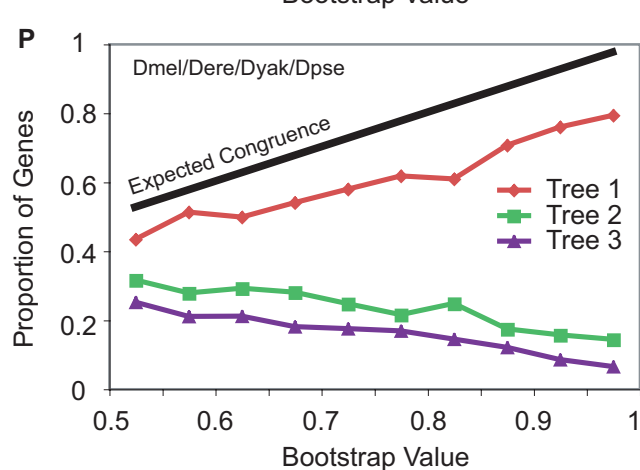

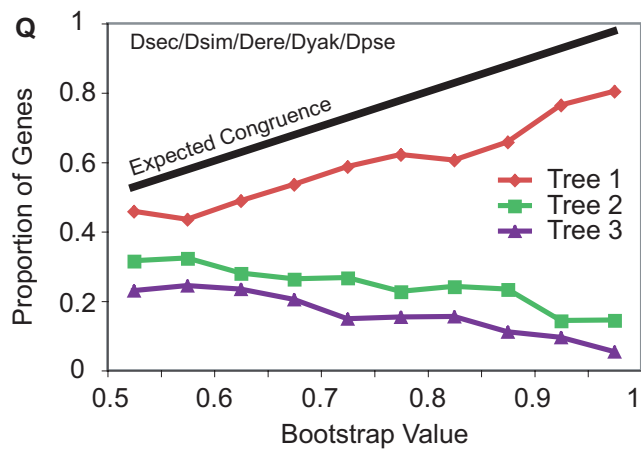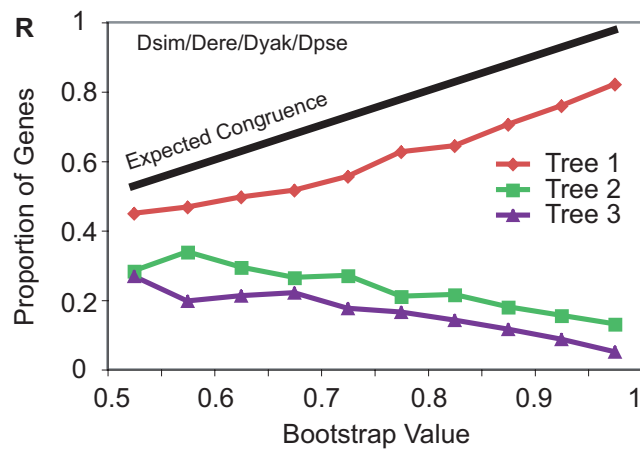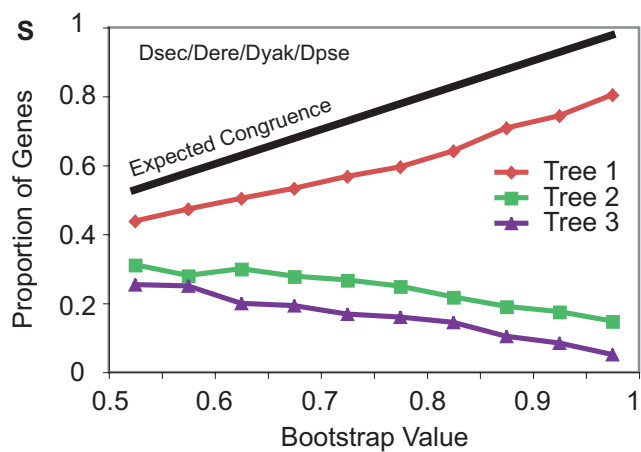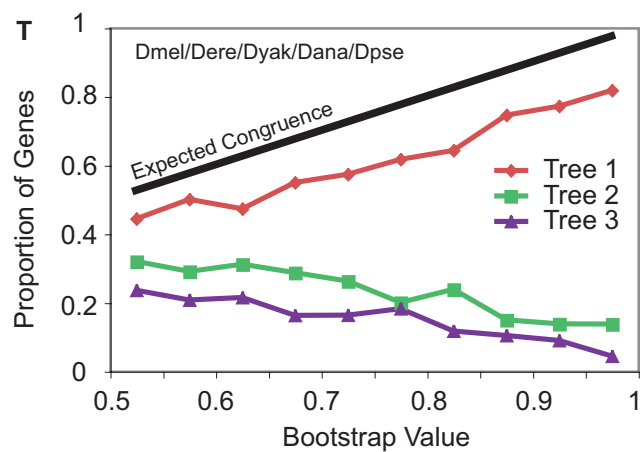

Supplement: Figure S2 — An excess of incongruence above what is expected by chance was observed using the HKY model for genes from Dmel, Dsec, Dsim, Dere, Dyak, Dana, and Dpse (A), Dmel, Dsec, Dsim, Dere, Dyak, and Dana (B), Dmel, Dsec, Dsim, Dere, Dyak, and Dpse (C), Dmel, Dsec, Dere, Dyak, Dana, and Dpse (D), Dmel, Dsim, Dere, Dyak, Dana, and Dpse (E), Dsec, Dsim, Dere, Dyak, Dana, and Dpse (F), Dsec, Dere, Dyak, Dana, and Dpse (G), Dmel, Dsim, Dere, Dyak, and Dana (H), Dsim, Dere, Dyak, Dana, and Dpse (I), Dmel, Dsec, Dere, Dyak and Dana (J), Dsim, Dere, Dyak, and Dana (K), Dsec, Dsim, Dere, Dyak, and Dana (L), Dsec, Dere, Dyak, and Dana (M), Dmel, Dsec, Dere, Dyak, and Dpse (N), Dmel, Dsim, Dere, Dyak, and Dpse (O), Dmel, Dere, Dyak, and Dpse (P), Dsec, Dsim, Dere, Dyak, and Dpse (Q), Dsim, Dere, Dyak, and Dpse (R), Dsec, Dere, Dyak, and Dpse (S), and Dmel, Dere, Dyak, Dana, and Dpse (T). Genes were binned by bootstrap value, and the proportion of genes supporting tree 1 (red line), tree 2 (green line), and tree 3 (purple line) were plotted. The expected congruence based on the bootstrap value in each bin (black solid line) demonstrates the excess incongruence. (68 KB PDF) [file pgen.0020173.sg002.pdf]
